# Autokeras Approach: A Robust Automated Deep Learning Network for Diagnosis Disease Cases in Medical Images

**DOI:** 10.3390/jimaging9030064

**Published:** 2023-03-08

**Authors:** Ahmad Alaiad, Aya Migdady, Ra’ed M. Al-Khatib, Omar Alzoubi, Raed Abu Zitar, Laith Abualigah

**Affiliations:** 1 Department of Computer Information Systems, Jordan University of Science and Technology, Irbid 22110, Jordan; 2 Department of Computer Sciences, Yarmouk University, Irbid 21163, Jordan; 3 Department of Computer Science, Jordan University of Science and Technology, Irbid 22110, Jordan; 4 Sorbonne Center of Artificial Intelligence, Sorbonne University-Abu Dhabi, Abu Dhabi P.O. Box 32092, United Arab Emirates; 5 Computer Science Department, Prince Hussein Bin Abdullah Faculty for Information Technology, Al al-Bayt University, Mafraq 25113, Jordan; 6 College of Engineering, Yuan Ze University, Taoyuan 320315, Taiwan; 7 Hourani Center for Applied Scientific Research, Al-Ahliyya Amman University, Amman 19328, Jordan; 8 Faculty of Information Technology, Middle East University, Amman 11831, Jordan; 9 Applied Science Research Center, Applied Science Private University, Amman 11931, Jordan; 10 School of Computer Sciences, Universiti Sains Malaysia, Pulau Pinang 11800, Malaysia

**Keywords:** artificial intelligence (AI), convolutional neural network (CNN), deep learning (DL), malaria parasites

## Abstract

Automated deep learning is promising in artificial intelligence (AI). However, a few applications of automated deep learning networks have been made in the clinical medical fields. Therefore, we studied the application of an open-source automated deep learning framework, Autokeras, for detecting smear blood images infected with malaria parasites. Autokeras is able to identify the optimal neural network to perform the classification task. Hence, the robustness of the adopted model is due to it not needing any prior knowledge from deep learning. In contrast, the traditional deep neural network methods still require more construction to identify the best convolutional neural network (CNN). The dataset used in this study consisted of 27,558 blood smear images. A comparative process proved the superiority of our proposed approach over other traditional neural networks. The evaluation results of our proposed model achieved high efficiency with impressive accuracy, reaching 95.6% when compared with previous competitive models.

## 1. Introduction

Malaria is a transmissible and life-threatening disease, as professed by the World Health Organization (WHO), and is prevalent in many countries, especially Senegal and those in Asia and Africa [1]. The primary cause of malaria is parasitesrecognized as Plasmodium, which infect human red blood cells (RBCs). Malaria is transmitted via mosquitoes, inside which the parasite lives. Once a mosquito bites a person, the person becomes infected with malaria [2]. In 2019, the WHO stated that 229 million cases of malaria were diagnosed, and in the same year, the number of deaths reached 409,000 [3].

It is worth noting in this report that the proportion of children under the age of five out of the total number of people who died of malaria reached 67% (274,000), which indicates that malaria is one of the most-significant causes of death among children in the world. In the last few years and during the COVID-19 pandemic, the number of malaria patients rose, which is a dire circumstance [3,4]. Therefore, malaria remains an acute health concern in large parts of the world, in particular in developing countries. As such, an automated diagnostic method that can decrease the time and cost of the recognition of malaria with good diagnostic performance becomes imperative.

In detecting malaria, thin and thick blood smears are usually taken from possibly infected people and examined on glass slides. These smears are viewed under a light microscope for surveillance. This diagnostic technique requires a high level of expertise to achieve precise results. Moreover, further complications regarding disease detection and medication probably lead to inconsistent and delayed results due to inadequate tools and proficiency in developing regions, as well as many other circumstances [5].

Artificial intelligence comprises machine learning and deep learning neural networks that could be used to handle current problems in healthcare around the world [6]. The current systems related to artificial intelligence in medicine already involve various tasks, from image segmentation to biometric measurement. However, the construction and training of a high-tech machine learning model require an in-depth understanding of the mathematical and engineering concepts of artificial intelligence, as well as choosing suitable algorithms and tuning the hyperparameters of the models. This is usually an arduous task for many proficient engineers and computer scientists, let alone healthcare specialists with restricted experience in computer science. Regarding malaria parasite detection using machine learning techniques, much work has been performed. For example, in [7], the authors used powerful classifiers (namely ResNet and DenseNet) via the transfer learning technique to classify cell images as either parasitized or uninfected. Another approach to tackling the problem of detecting malaria in blood images can be found in [8], where the work was based on different machine learning methods (decision tree, support vector machine, naïve Bayes, and K-nearest neighbor), which were fit on six features extracted by the VGG16, VGG19, ResNet50, ResNet101, DenseNet121, and DenseNet201 models. Such a manual approach to machine learning for cell image classification of malaria requires experts in machine learning to prepare the model. However, the works [7,8], as well as the greatly different works on machine learning (ML) adopted for the malaria detection problem [9] suffer from different issues: the first one is that human effort is required in order to extract the most-valuable features to feed the classifiers efficiently. Furthermore, this approach requires specialists in ML and DL to build a robust model, in addition to choosing the best hyperparameters of the model to distinguish the malaria images from the normal ones. Therefore, AutoML Vision (Autokeras) could be a possible solution to mitigate these issues, as it has a highly friendly user interface. AutoML Vision offers a highly automated model improvement framework to help people with little computer encoding knowledge or experience build and train their own machine learning models.

**Figure 1:**
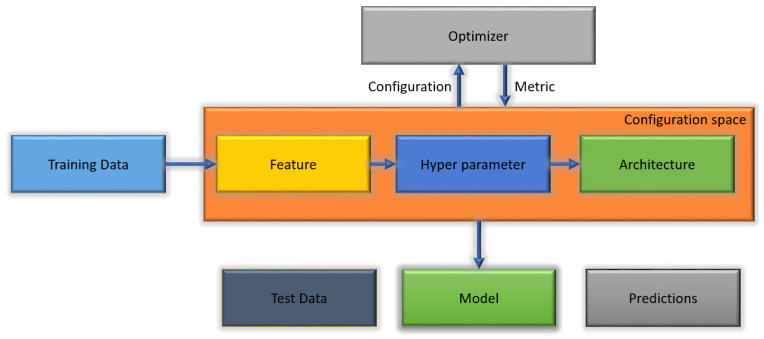
Auto-machine learning technique.

The automated machine learning methods to detect malaria reduce the human effort in the construction of networks as an alternative to creating classifier networks from scratch and modifying the parameters. This work was based on a novel technique that automatically discovers the best deep learning model to classify cells into parasitized or healthy cells. No experience in coding is required. It depends on a few lines of code to function. Large technology companies have made a respectable effort to disseminate various automated machine learning software, such as Google Cloud AutoML and Azure automated machine learning. The benefit of such services is the ability to build high-performance models with as few experts in machine learning as possible. Moreover, several of the open-source software frameworks also achieve high performance, for example AutoWeka [10], AutoSKLEARN [11], Autokeras [12], and Teapot. Unfortunately, the above-mentioned services are restricted when applied. For instance, they are suited only to a specific problem, e.g., natural language processing (NLP), image classification, or speech recognition [13]. Figure 1 illustrates the AutoML algorithm.

We sought in this paper to study the efficiency and performance of auto-machine learning systems in the medical field. As far as we know, this is the first paper to test the power of auto-machine learning (AutoML) by implementing Autokeras, an automated deep learning network, to classify cell images as infected or not with the malaria parasite. Autokeras’s deep learning model was preferred over other approaches to automatic machine learning for several reasons mentioned in Section 3.1. The following points describe the main contributions of this research:

- This work represents a pioneering work in classifying images of malaria-infected and normal blood cells by the AutoKeras software, one of the auto-machine learning systems.
- We propose preprocessing on malaria image datasets before applying the Autokeras model.
- We chose the best model out of 20 trials performed by the Autokeras software to search for the best network that gave the lowest validation loss.
- We demonstrated the high performance of the Autokeras software in detecting malaria-infected cells and its superiority over the traditional deep learning (DL) models, which require machine learning experts.

The results of the proposed method were applied to various medical problems, and the results were compared to other methods. The proposed method obtained better results in solving the test problems than the other methods selected from the literature.

The remainder of this paper is organized in the following manner. Section 2 lists the main related works in the literature that present the malaria diagnosis paradigm. The adapted Autokeras model is introduced in Section 3. Then, the evaluation metrics used are illustrated in Section 4. Our proposed methodology is discussed with the details of the implementation in Section 5. Lastly, the work is concluded with an outlook on future works in Section 6.

## 2. Related Work

In this section, the related works that have used deep learning and machine learning techniques are presented as follows. The immense capability of convolution neural networks (CNNs) in image detection in various fields has been demonstrated. Several studies have been conducted on malaria parasite detection, which can be summarized as follows. In [14], the authors used a deep CNN to automatically discover malaria in thin blood smear images by proposing an entire computer-aided diagnosis structure. In order to optimize the process of feature selection, they used the transfer learning technique. By using the feature matrix in the intermediate layers, the layer embedding was removed from the intermediate convolutional layers as an additional layer of security. The proposed transfer learning technique exploited the ResNet 152 network combined with the deep greedy model for fitting.

In [15], the study was based on the proposed data augmentation convolutional neural network (DACNN) deep learning model, which used the reinforcement learning technique to solve such issues. They compared the effectiveness of their suggested DACNN with others: CNN and directed acyclic graph convolutional neural network (DAGCNN). By the experiment’s test result, they proved that their DACNN outperformed previous works in treating and classifying the images, where the DACNN obtained a 94.79% accuracy.

In [8], the authors proposed an approach based on using the features of ResNet50, ResNet101, VGG16, VGG19, DenseNet121, and DenseNet201. Then, they used machine learning models based on decision tree, support vector machine, naïve Bayes, and K-nearest neighbor to identify the malaria parasite in the cell images. The results showed that their proposed model could successfully detect the disease in the dedicated dataset with an accuracy of 94%.

In [16], the PlasmodiumVF-Net framework was introduced to determine whether an image of a patient sample showed infection. If malaria infection was confirmed, another classification was performed to specify if the individual was infected with Plasmodium falciparum or Plasmodium vivax. The work was grounded on the mask regional convolutional neural network (Mask RCNN) and the ResNet50 classifier. They used a dataset containing 6000 images, which they made publicly available. Their framework achieved an accuracy of 90%.

In [17], the authors used a transfer learning approach to identify images of malaria parasite infection by integrating the current Visual Geometry Group (VGG) network and support vector machine (SVM). This hybrid approach was executed by training the topmost layers and freezing the remaining layers. First, the “k” layers of the VGG model were reserved, and the (n − k) layers were replaced bySVM. Finally, the experimental results showed that this combination of VGG19 and SVM achieved a classification accuracy of 93.1%

The work [18] was based on the benefit of a pretrained deep convolutional neural network algorithm for the detection of malaria in images. They achieved a detection accuracy of 93.89% and 95.20% by using the GoogLeNet and ShuffleNet V2 models, respectively. Moreover, they proved that the ShuffleNet V2 model was three-times faster than GoogLeNet in the training.

In [19], the authors built a DBN to classify 4100 blood smear images into the classes: parasite or not. The suggested DBN was built utilizing the contrastive divergence method for pretraining by stacking limited Boltzmann machines. They found that stacking 800 layers could achieve a 96.32% accuracy. According to the imbalanced dataset used in this paper (669 parasite and 3431 non-parasite), the F1-metric, which was best suited to the performance evaluation, reached 89.66%.

Another approach using in an imbalanced dataset can be found in [9], where the work was based on building a convolutional neural network. The authors aimed to predict the existence of malaria-infected cells using images obtained by microscopy of thin and thick peripheral blood smears. They also used a transfer learning model to compare the proposed model against the pretrained models. They achieved an accuracy, precision, and sensitivity of 96.97%, 97.00%, and 97.00%, respectively.

A computer-aided design was proposed in [20] to recognize malaria infections from blood images obtained by microscopy. The bilateral filtering process was used in the suggested method to eliminate the noise and increase the image quality. The image processing techniques of adaptive thresholding and morphological operations were utilized to discover the malaria infection inside an individual cell. Their method achieved a detection accuracy greater than 91%.

In [21], the work was based on suggesting a customized CNN. The authors exploited image augmentation techniques and bilateral filtering to extract the features of red blood cells before passing them to the model for training. According to the data augmentation techniques, the number of data was increased from 27,558 to 173,700 images. Therefore, the model was expected to be more generalized and more accurate (accuracy equal to 96.82).

The work in [22] adopted the problem of diagnosing malaria infection from blood cell images. To tackle the problem, the authors suggested a multiheaded-attention-based Transformer model. In order to illustrate the efficiency of the model, they used the gradient-weighted class activation map (Grad-CAM) technique. This technique is responsible for recognizing the parts of an image that need much more attention than the others.

In [23], the authors’ proposed model involved three convolutional dense layers and one fully connected layer. The neural network was a sequence of multiple convolutional layers using several existing filters in the layers, resulting in a reasonable accuracy. Model training was implemented, and several blood image datasets served to assess their proposed model’s accuracy. The CNN was implemented using restricted computational resources, resulting in an accuracy of 95%.

The authors of [24] used the EfficientNetB0 model to classify blood cell images as infected or not. To decrease the time and boost the original feature sets, their work was based on fine-tuning (transfer learning). Their proposed EfficientNetB0 model obtained an accuracy of 94.70% after training for fifty epochs. As stated in their work, they verified that the transfer learning of the EfficientNetB0 model could obtain good accuracy in recognizing malaria-infected cells in blood smear images without requiring preprocessing, data augmentation, or other techniques.

The work in [25] was based on proposing a convolutional neural network (CNN). The construction of this CNN involved 20 layers, which was essentially required to differentiate between infected cell images and healthy ones. Their work was based on using the same dataset (LHNCBC) that we used in this study, which contains 27,558 cell images. They achieved a 95.28% overall accuracy in the experimental test result.

The proposed work by [26] relied on two phases. The first phase used a graphical user interface to detect infected and fragmented red blood cells. The second phase determined whether the thin blood smearcell images presented infection, for which a feed-forward neural network (FNN) was proposed. The dataset used in this paper consisted of 27,560 benchmark images. Their proposed technique achieved a 92% accuracy in the testing phase.

However, not all current deep learning (DL) models can be readily implemented for malaria diagnosis systems, as they require extra effort and may be considered as a technical challenge, as in the previous solutions of deep learning works. Moreover, constructing a high-efficiency DL model for a particular task can face issues that make these processes more complex, such as being resource-expensive, time-consuming, and depending heavily on human experience through a trial-and-error method. To this end, Autokeras provides a promising alternative to the manual DL solutions and requires little effort or involvement of the user, as it excels in providing the best-performing deep learning network along with the feature engineering (mining, choosing, and building) and network construction (hyperparameter choice and fine-tuning).

## 3. Model Development

### 3.1. Auto-Machine Learning

**Figure 2:**
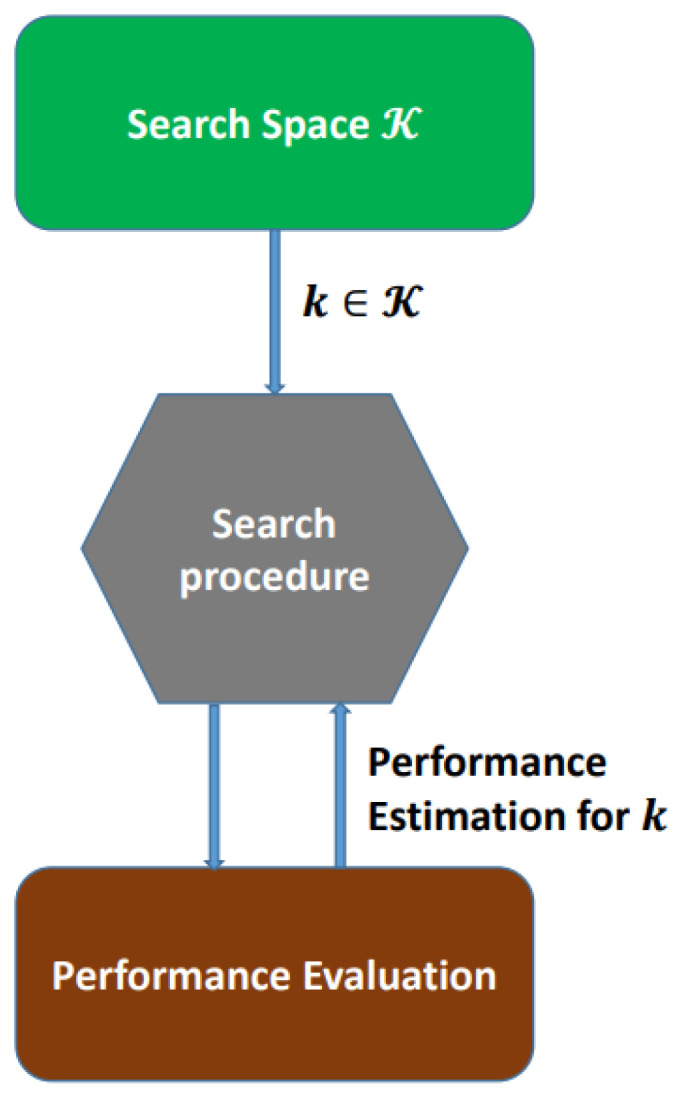
NAS procedure.

The AutoML technique can be described as automatically discovering the best classifier for a certain dataset. As such, performing this method on neural networks results in identifying the model’s structure and the hyperparameters required to train the classifier. It relies on a neural architecture search algorithm (NAS). Figure 2 gives the NAS procedure. It has a specific number of trials provided by the user to search for and choose the most-powerful architecture and parameters of the neural network (NN) [27].

However, the basic problem that the NAS aims to solve is as follows: Assuming a search space *K* for the NN architecture, *D* as the input data, which are split into training data $D^{T}$ and validation data $D^{V}$, and finally, the cost function *C*, the main goal of the NAS technique is to detect the best neural network $k^{*}\in K$ that efficiently obtains the minimum cost value over the dataset *D*. This corresponds to finding the $k^{*}$ fulfilling

(1) $$\underset{k^{*}\in K}{\arg\min}\;C\left(k\left(\theta^{*}\right),D^{V}\right)\;$$

*C* is the metric assessment function such as the accuracy and mean-squared error (MSE). The following formulas explain the mathematical notations for each one:

(2) $$acc\left(\%\right)=\frac{\min(x,\hat{x})}{\max(x,\hat{x})}\times 100\;$$

(3) $$MSE=\frac{\sum {\left(x-\hat{x}\right)}^{2}}{n}\;$$

in which *x* is the truevalue, $\hat{x}$ is the forecast value, and *n* is the size of the data.

We can identify θ as the learnable parameters of *k*, and the following equation explains it:

(4) $$\theta^{*}=\underset{\theta^{*}\in K}{\arg\min}\;L\left(k\left(\theta \right),D^{T}\right)\;$$

The search space *K* includes all the neural architectures that can be derivedfrom the startingarchitectures. The searcher unit is responsiblefor the searching process in the neural architecture search (NAS) algorithm.

In our work, we applied the Autokeras technique as our model. However, there are different algorithms for AutoML such as Autokeras [12], Auto-Sklearn [11], and AutoWEKA [10]. The following two subsections provide an overview plus the architecture of the utilized technique; moreover, it presents why we chose this approach over the other techniques.

### 3.2. Autokeras Overview

Autokeras is an open-source software library established by DATA Lab at Texas A&M University [12]. The development of the Autokeras software requires Python programming language Version 3 with the Keras library.

The main advantage of using the Autokeras software on a local machine is the ability to dispense with building Dockers and Kubernetes in the cloud. Unlike other AutoML systems such as TPOT [28], SMAC [29], Auto-Sklearn [11], and AutoWEKA [10], which focus on shallow models, Autokeras focuses on deep learning models. The primary motivation for using the Autokeras service can be summarized as three points as follows:

- Cloud services are paid services, which may be a problem for many users who intend to use AutoML algorithms, unlike Autokeras, which is free.
- Usually, cloud-based AutoML requires users to have a good background in computer science, which is the opposite for Autokeras with its ease of use.
- The Autokeras services are characterized by their availability to all people, which can be used locally on a personal desktop. As such, the Autokeras services solve the problem of the security issues associated with using other AutoML applications.

### 3.3. Autokeras Architecture

**Figure 3:**
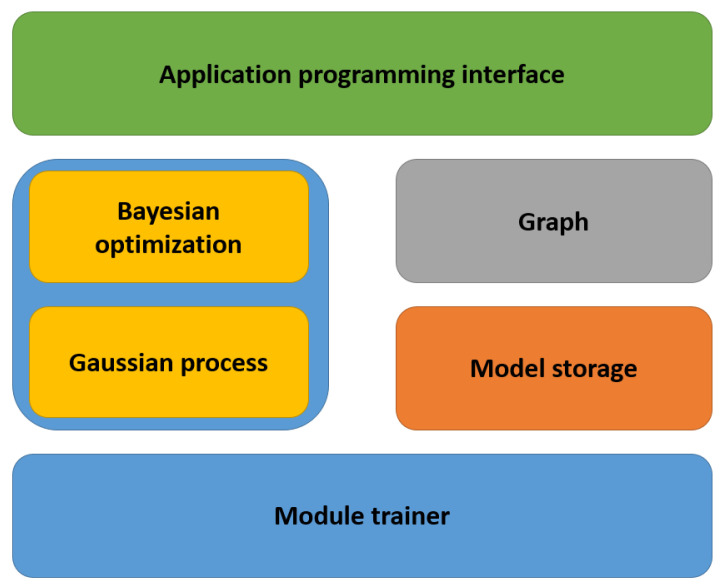
Autokeras system architecture. 1. Initially, the API is called by the user. 2. Then, the Searcher creates NN architectures on the CPU. 3. The graph moduleconstructs the actual neural networks with the parameters on the RAM from the NN architectures. 4. Following this, a copy of the NN is sent to the GPU for training. 5. Finally, the fit NNs are kept on the storage devices, and an update is applied to the searcher according to the training outcomes. Repeat Steps 2 to 5 until the time limit is reached.

Figure 3 shows the Autokeras system architecture [12]. As described in Figure 3, the architecture of Autokeras preservesthe full resourcesof both the CPU and GPU, and it effectively uses the memory by only storing the currently utilized data in the RAM. At the same time, the remaining information is saved on storage devices, for example hard drives. Firstly, the API appears at the top, which the user is responsible for calling. The API callsthe modules of themiddle level in order to perform specific functionalities. The searcher Autokeras algorithm uses Bayesian optimization as a guide in the neural architecture search. Such a searcher is responsible for controlling the operations of the network morphing by containing the Gaussian process and Bayesian optimizer, which run on the CPU.

Secondly, the model trainer unit is used for the GPU computation. The model trainer is responsible for achieving parallelism by training a particular neural network with the training data in a separate process. The graph module processes the neural network’s computational graphs. The current neural architecture in the graph module is located on the RAM to speed up the access process. The stored models are considered as a trained model collection. Due to the enormous size of the neural networks, in addition to the difficulty of storing them in the memory, all the trained models are savedon the storage devices.

## 4. Model Evaluation

The confusion matrix (CM) (an error matrix) can be defined as a concise table or tool that reveals how well the model can predict from a particular testing dataset. A CM consists of rows and columns giving the labels of the ground truth and the predicted class. The ground truth is the actual infected and uninfected blood cells. On the other hand, the predicted values specify the number of correct and incorrect classifications made by the model. The evaluation measures used in the confusion matrix are presented as follows:

- True positives (TPs) signify the amount of correctly predicted positive samples.
- True Negatives (TNs) identify the number of correctly predicted negative samples.
- False Positives (FPs) are cases where the images were predicted as positive, but were not
- False Negatives (FNs) are cases where the images were predicted as negative, but were not.

Accuracy is a measure of all correct predictions from all the samples sets and is determined by Equation (5). We calculated the model’s accuracy, precision, recall, and F1-score as the metrics. The following illustrates the concepts and the equation for each metric.

(5) $$Accuracy=\frac{TP+TN}{TP+FP+TN+FN}$$

Precision is the number of times the model made a correct prediction out of an actual class, determined by Equation (6).

(6) $$Precision=\frac{TP}{TP+FP}$$

The recall is known as the true positive rate (TPR) or the sensitivity. It implies the percentage of accurately predicted infected cells or TPs to all TPs and FNs and is determined by Equation (7).

(7) $$Recall=\frac{TP}{TP+FN}$$

The F1-measure is a weighted average between the recall and precision, which can have a maximum score of 1, which is the best case, and the lowest score of 0, and it is determined by Equation (8).

(8) $$F-measure=\frac{2\times Recall\times Precision}{Recall+Precision}$$

## 5. Methodology

### 5.1. Dataset

**Figure 4:**
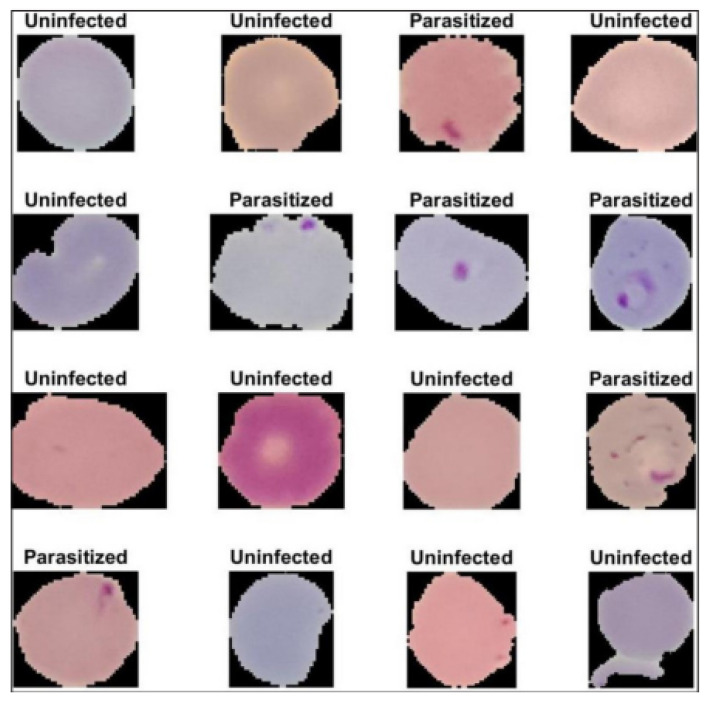
Sample of uninfected and infected cell images.

In this work, the publicly available malaria dataset provided by Lister Hill National Centre for Biomedical Communications (LHNCBC) [30] was used to evaluate our experimental test result. The dataset was manually gathered and classified by experts. The dataset images were taken at the Bangladesh hospital where the Giemsa-stained thin blood smears were taken from 200 persons (50 healthy persons and 150 P. falciparum-infected patients). The dataset contains 27,558 cell images with identical amounts of infected and uninfected cells: 13,779 parasitized and 13,779 uninfected. Figure 4 shows sample images of uninfected and infected cells [25].

### 5.2. Experimental Platform

In this work, the Windows 10 system platform with 64 bits was used as the software platform for implementing the experiments of this research, where the hardware consisted of 2 GB VRAM with NVIDIA GeForce MX13, and the Intel(R) also was used as a hardware component with Core(TM), 12 GB RAM, and i5 = 8250U CPU @ 1.60–1.80 GHz. The experiments were performed in the environment of a Jupyter Notebook with Python programming language Version 3.9.

### 5.3. Preprocessing

Any model’s performance is entirely governed by the data that are fed to it. Therefore, data preprocessing plays a primary role in conducting tests. Therefore, in this work, some preprocessing steps were conducted on the data, such as resizing the images to 32 (to reduce time), converting them to arrays (because the input data in the Autokeras function are expected to be an array), and then shuffling all the training data.

### 5.4. Implementation

**Figure 5:**
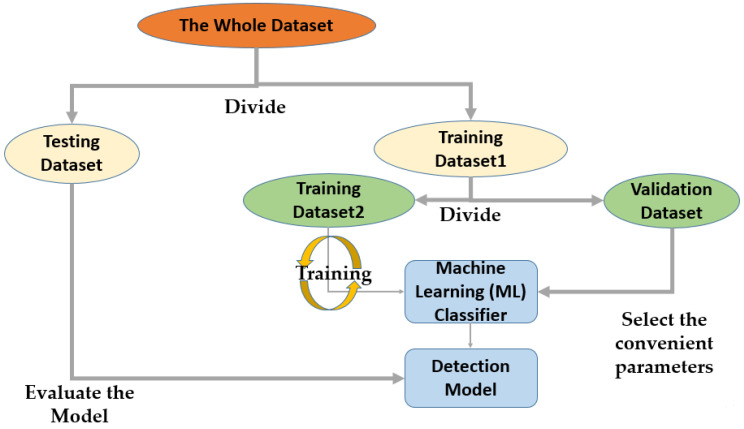
“Hold-Out” validation technique.

In this paper, the “Hold-Out” validation mechanism [31] was adopted for the experimental test. Figure 5 shows the procedure of the “Hold-Out” validation mechanism. The dataset was divided into training, testing, and validation sets at a ratio of 56:30:14, respectively 15,432, 8268, 3858 images, before the model began training.

Table 1 presents a complete description of the data division. The main goal of this division of the training and testing data was to build an accurate prediction model. Furthermore, the main objective of a small (%) validation dataset was to keep the model from overfitting and make it more precise on the unseen dataset.

In detail, once the data were ready for training, the Autokeras system started locally working with a workflow as follows. The API conveys the dataset after preprocessing it to the searcher to begin the search process. We trained the Autokeras software with a max of 20 trials to enable the searcher to find the best network architecture with the lowest validation loss. The Bayesian optimizer creates a new CPU architecture in such a searcher. The mission of the Bayesian optimizer is to call the graph module to construct the created neural architecture into an actual neural network in the RAM. The novel neural architecture is used to copy the model trainer to GPU for training the dataset. Feedback on the model’s performance is passed to the searcher to make any necessary modifications to the Gaussian process.

## 6. Result and Evaluation

**Figure 6:**
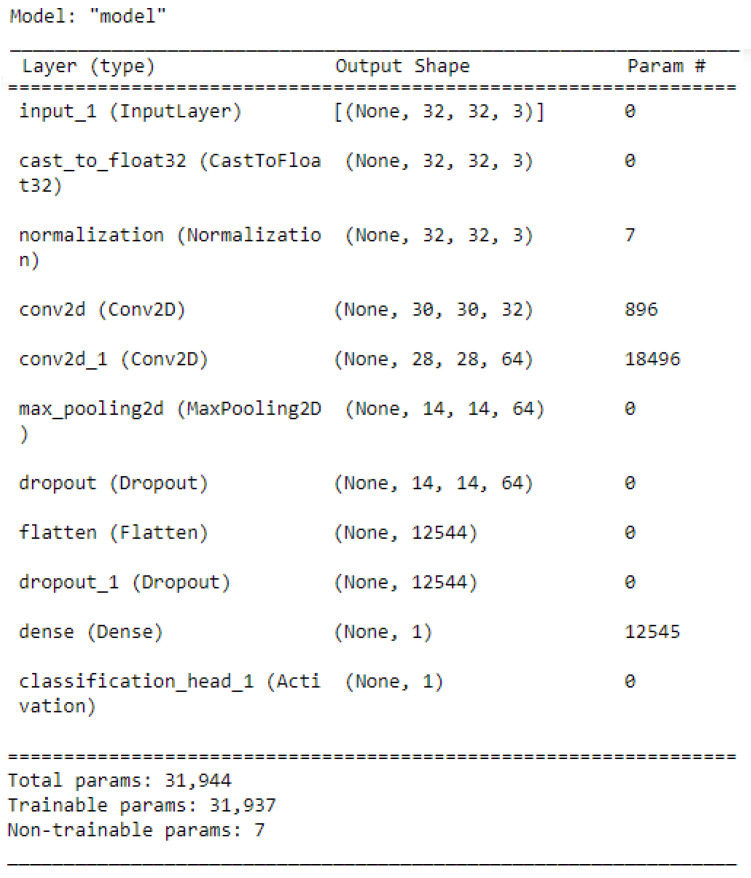
The best network architecture provided by the AutoKeras model from 20 trials.

**Figure 7:**
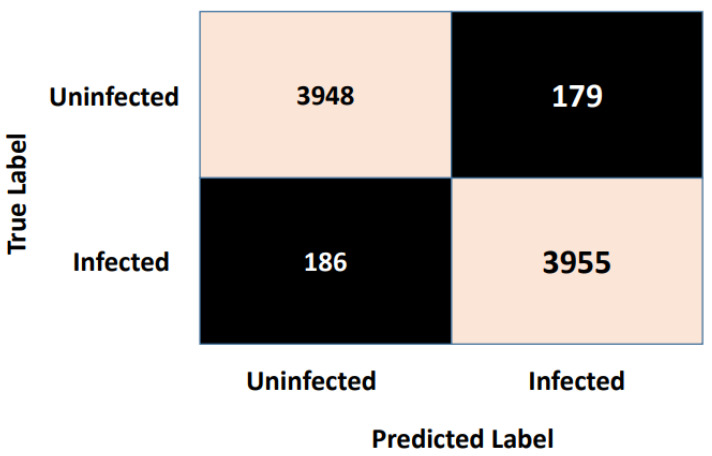
Confusion matrix of the malaria dataset.

As we mentioned earlier, the metrics used to assess the performance of the Autokeras model were the accuracy, precision, recall, and F1-measure, which are illustrated in detail in Section 6. Figure 6 illustrates the best network architecture, which showed high classification performance provided by the AutoKeras model from 20 trials, which is the number of networks assessed by AutoKeras (i.e., 20 trials = 20 networks). This highest-performance network was saved and used for the prediction task. Once the prediction task was accomplished, the confusion matrix metrics were used to assess the performance of the best Autokeras model. Figure 7 shows the confusion matrix. As is clear from Figure 7, the vertical axis indicates the target class (actual label), while the system’s predicted class (output label) is presented along the horizontal axis.

**Figure 8:**
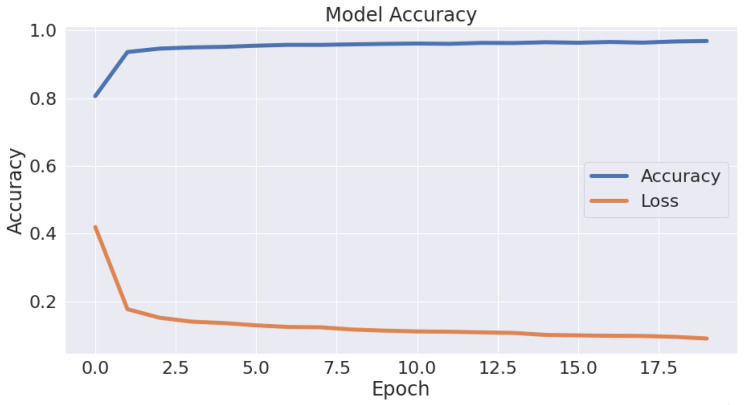
The accuracy vs. the loss during the validation phase.

**Figure 9:**
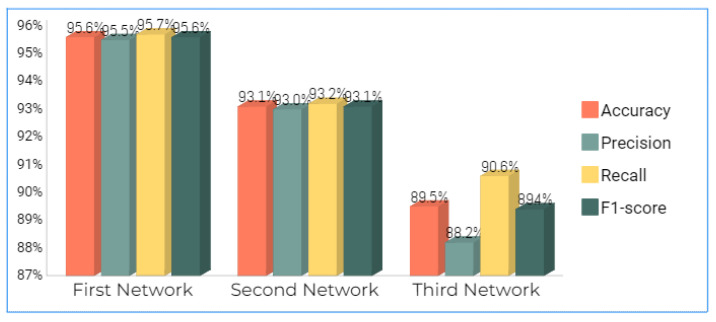
The metrics’ results for the three best networks obtained after 20 AutoKeras trails.

These four metrics aimed to better describe the adopted model’s evaluation results, which can be derived from the confusion matrix. A complete review of the performance evaluation metrics is offered in Table 2. As is clear, the Autokeras model had a promising ability to distinguish between the images of the infected thin blood smear cells from the uninfected ones with an impressive accuracy of 95.6%, precision of 95.5%, recall of 95.7%, and F1-score of 95.6%. The accuracy and loss schemes during the 20 epochs are illustrated in Figure 8. It is clear that a 95.6% accuracy was obtained after ten iterations. Furthermore, we show the evaluation metrics’ results for the three best networks obtained after 20 AutoKeras trials in Figure 9. As is clear from the figure, we adopted the first network for our prediction results over the malaria testing dataset.

Despite the large size of the malaria dataset of the images of the thin blood smear cells, Autokeras’ proposed model proved its robustness in classifying the cell images into infected or uninfected with malaria.

**Figure 10:**
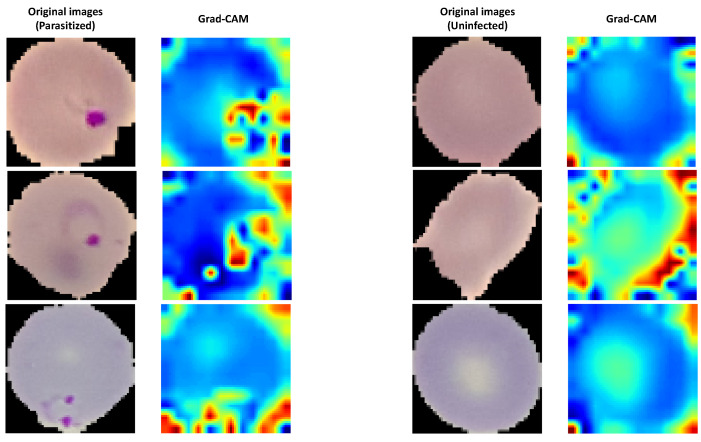
Process of the Grad-CAM algorithm for the original image via the AutoKeras model.

The Grad-CAM algorithm was applied in order to show a visualized localization of prominent features appearing on the parasitized blood cell. This approach helped add transparency to our outcomes. Grad-CAM is a generic method that uses a CNN as the final activation layer. As such, a set of high-level features is produced in the shape of a heat map. In Figure 10, the parasite detection using the Grad-CAM algorithm is presented for the proposed model. As is clear from the figure, the proposed model kept its attention on the critical areas. Moreover, it produced the most-compelling interpretation and -precise revelation of the parasites, as the hot region better preserved the infection’s localization.

Table 3 introduces a complete comparison between the results using the Autokeras model against the results of the other related works. Five benchmarks were used to evaluate the prediction precision for each model, specified as follows: the publication title, publication year, number of images in the dataset used, the approaches used, and the overall accuracy. The good accuracy, which was beyond 90% for all the models, indicates each model’s efficiency in the malaria classification tasks.

As is clear in Table 3, the Autokeras model outperformed the previous works in recognizing malaria disease from images of blood smear cells. Moreover, the adopted Autokeras model proved its advantages over some works, such as those in [8,15], which were performed on the same dataset, with an accuracy of 94.79% and 94%, respectively. It is important to note that some works such as [19] produced more accurate models, for which they achieved 96.32%. However, it is worth knowing that they used an imbalanced dataset. Therefore, accuracy is not the best metric in such a case. Therefore, the F1-score can be a more suitable metric. In [19], their model produced an F1-score equal to 89.66%.

Moreover, the papers in [9,21] demonstrated better performance than ours due to the augmentation techniques. Therefore, the size of the dataset increased, which may help the model generalize better (more data result in a more accurate model [32]). In our work, we did not augment the dataset, which mimics the real-world scenario. Furthermore, the proposed work can produce a robust model that can achieve approximately 1 percent less than their works.

In conclusion, this paper applied a novel optimization technique to tune the hyperparameters of the machine learning techniques, called automated machine learning. The proposed method proved its ability to obtain the optimal parameter values, which better reflect the results of the utilized evaluation measures compared to the other methods from the literature.

## 7. Conclusions

This work adopted an open-source automatic AutoKeras deep learning model to identify malaria parasites in blood cell images. AutoKeras focuses on techniques for automatically determining the highest-performing model for a given dataset. This theme has received attention recently and has been developing quickly ever since. However, the primary motivation for automating the hyperparameters’ tuningin any model is to find an automatic ML solution that can achieve a high degree of performance accuracy and lead to an innovative level of automation in the future using artificial intelligence.

The proposed Autokeras malaria classification approach achieved the best overall accuracy of 95.6%. It was evident from the experimental test implemented that the robustness of Autokeras outperformed previous works from some studies. This proved that the proposed method can solve other similar problems efficiently according to the results of the comparisons with the the-stat-of-the-art methods.

Other machine learning methods combined with advanced optimization algorithms can be used to solve the hyperparameter problems in any model and find a robust automatic ML method. Moreover, other applications can be addressed using the proposed method, such as brain medical images, patient risk identification, pancreatic cancer, sarcoma, pattern imaging analytics, stomach cancer, clinical trial research, predicting epidemics, kidney cancer, maintaining healthcare records, and others.

The limitations of using auto-machine learning are that it requires much time to train with such a limited platform and requires a faster device. We recommend using a faster device in such cases.

## Figures and Tables

**Table 1:** The total number of images after the division of the data into training, validation, and testing sets.

Class	Training Dataset	Validation Dataset	Testing	Fraction	Total Images
Uninfected cell images	7716	1929	4134	50%	13,779
Infected cell images	7716	1929	4134	50%	13,779
Fraction	56%	14%	30%		
Total	15,432	3858	8268	100%	27,558

**Table 2:** Evaluation metrics’ results.

Accuracy	Precision	Recall	F1-Measure
95.6%	95.5%	95.7%	95.6%

**Table 3:** Comparison between the Autokeras model and previous works.

Refs.	Number of Images	Models	Accuracy
[25]	27,558	CNN	95.28%
[18]	-	ShuffleNet V2	95.20%
[19]	4100	DBN	96.32%
[9]	714	CNN	96.97%
[20]	27,558	Custom algorithm using bilateral filtering	91%
[21]	27,558	CNN	96.82
[15]	27,558	DACNN	94.79%
[24]	27,558	EfficientNetB0	94.70%
		Naïve Bayes	
[8]	27,558	Support vector machine	94%
		K-nearest neighbor	
[16]	6000	ResNet50	90%
		ResNet50	
[26]	27,560	Feed-forward neural network (FNN)	92%
The proposed model	27,558	Autokeras	95.6%

## Data Availability

Data is available from the authors upon reasonable request.

## References

[1] WHO (2019). Global Perspectives on Assistive Technology: Proceedings of the GReAT Consultation 2019.

[2] Rahman A., Zunair H., Rahman M.S., Yuki J.Q., Biswas S., Alam M.A., Alam N.B., Mahdy M. (2019). Improving malaria parasite detection from red blood cell using deep convolutional neural networks. arXiv.

[3] WHO (2019). World Malaria Report 2019.

[4] Chiodini J. (2020). Online learning in the time of COVID-19. Travel Med. Infect. Dis..

[5] Osei-Yeboah J., Kwame Norgbe G., Yao Lokpo S., Khadijah Kinansua M., Nettey L., Allotey E.A. (2016). Comparative performance evaluation of routine malaria diagnosis at Ho Municipal Hospital. J. Parasitol. Res..

[6] Ali R., Hardie R.C., Narayanan B.N., Kebede T.M. (2022). IMNets: Deep learning using an incremental modular network synthesis approach for medical imaging applications. Appl. Sci..

[7] Krishnadas P., Sampathila N. Automated detection of malaria implemented by deep learning in PyTorch. Proceedings of the 2021 IEEE International Conference on Electronics, Computing and Communication Technologies (CONECCT).

[8] Abubakar A., Ajuji M., Yahya I.U. (2021). DeepFMD: Computational Analysis for Malaria Detection in Blood-Smear Images Using Deep-Learning Features. Appl. Syst. Innov..

[9] Uzun Ozsahin D., Mustapha M.T., Bartholomew Duwa B., Ozsahin I. (2022). Evaluating the performance of deep learning frameworks for malaria parasite detection using microscopic images of peripheral blood smears. Diagnostics.

[10] Thornton C., Hutter F., Hoos H.H., Leyton-Brown K. Auto-WEKA: Combined selection and hyperparameter optimization of classification algorithms. Proceedings of the 19th ACM SIGKDD International Conference on Knowledge Discovery and Data Mining.

[11] Feurer M., Klein A., Eggensperger K., Springenberg J., Blum M., Hutter F. (2015). Efficient and robust automated machine learning. Advances in Neural Information Processing Systems 28 (NIPS 2015).

[12] Jin H., Song Q., Hu X. Auto-keras: An efficient neural architecture search system. Proceedings of the 25th ACM SIGKDD International Conference on Knowledge Discovery & Data Mining.

[13] Hibayesian GitHub—Hibayesian/Awesome-Automl-Papers: A Curated List of Automated Machine Learning Papers, Articles, Tutorials, Slides and Projects. https://github.com/hibayesian/awesome-automl-papers.

[14] Dey S., Nath P., Biswas S., Nath S., Ganguly A. (2021). Malaria detection through digital microscopic imaging using Deep Greedy Network with transfer learning. J. Med. Imaging.

[15] Oyewola D.O., Dada E.G., Misra S., Damaševičius R. (2022). A Novel Data Augmentation Convolutional Neural Network for Detecting Malaria Parasite in Blood Smear Images. Appl. Artif. Intell..

[16] Kassim Y.M., Yang F., Yu H., Maude R.J., Jaeger S. (2021). Diagnosing Malaria Patients with Plasmodium falciparum and vivax Using Deep Learning for Thick Smear Images. Diagnostics.

[17] Vijayalakshmi A., Rajesh K.B. (2020). Deep learning approach to detect malaria from microscopic images. Multimed. Tools Appl..

[18] Diyasa I.G.S.M., Fauzi A., Setiawan A., Idhom M., Wahid R.R., Alhajir A.D. Pre-trained deep convolutional neural network for detecting malaria on the human blood smear images. Proceedings of the 2021 International Conference on Artificial Intelligence in Information and Communication (ICAIIC).

[19] Bibin D., Nair M.S., Punitha P. (2017). Malaria parasite detection from peripheral blood smear images using deep belief networks. IEEE Access.

[20] Fatima T., Farid M.S. (2020). Automatic detection of Plasmodium parasites from microscopic blood images. J. Parasit. Dis..

[21] Maqsood A., Farid M.S., Khan M.H., Grzegorzek M. (2021). Deep malaria parasite detection in thin blood smear microscopic images. Appl. Sci..

[22] Islam M.R., Nahiduzzaman M., Goni M.O.F., Sayeed A., Anower M.S., Ahsan M., Haider J. (2022). Explainable Transformer-based deep learning model for the detection of malaria parasites from blood cell images. Sensors.

[23] Shah D., Kawale K., Shah M., Randive S., Mapari R. Malaria parasite detection using deep learning:(Beneficial to humankind). Proceedings of the 2020 4th International Conference on Intelligent Computing and Control Systems (ICICCS).

[24] Montalbo F.J.P., Alon A.S. (2021). Empirical Analysis of a Fine-Tuned Deep Convolutional Model in Classifying and Detecting Malaria Parasites from Blood Smears. KSII Trans. Internet Inf. Syst. (TIIS).

[25] Irmak E. (2021). A novel implementation of deep-learning approach on malaria parasite detection from thin blood cell images. Electrica.

[26] Manning K., Zhai X., Yu W. (2021). Image analysis and machine learning based malaria assessment system. Digit. Commun. Netw..

[27] Perez J.G.M. (2019). Autotext: AutoML for Text Classification. Master’s Thesis.

[28] Olson R.S., Bartley N., Urbanowicz R.J., Moore J.H. Evaluation of a tree-based pipeline optimization tool for automating data science. Proceedings of the Genetic And Evolutionary Computation Conference.

[29] Hutter F., Hoos H.H., Leyton-Brown K. Sequential model-based optimization for general algorithm configuration. Proceedings of the International Conference on Learning and Intelligent Optimization.

[30] LHNCBC LHNCBC Full Download List. https://lhncbc.nlm.nih.gov/LHC-downloads/downloads.html.

[31] Ripley B.D. (2007). Pattern Recognition and Neural Networks.

[32] Cho J., Lee K., Shin E., Choy G., Do S. (2015). How much data is needed to train a medical image deep learning system to achieve necessary high accuracy?. arXiv.

